# Fabrication and Characterization of Polymeric Pharmaceutical Emulgel Co-Loaded with Eugenol and Linalool for the Treatment of *Trichophyton rubrum* Infections

**DOI:** 10.3390/polym13223904

**Published:** 2021-11-11

**Authors:** Muhammad Abdullah Akram, Barkat Ali Khan, Muhammad Khalid Khan, Ali Alqahtani, Sultan M. Alshahrani, Khaled M. Hosny

**Affiliations:** 1 Drug Delivery and Cosmetics (DDC) Laboratory, Gomal Center of Pharmaceutical Sciences, Faculty of Pharmacy, Gomal University, Dera Ismail Khan 29050, Pakistan; scisp2@aliyun.com (M.A.A.); Barkat.khan@gu.edu.pk (B.A.K.); 2 Department of Pharmacology, College of Pharmacy, King Khalid University, Abha 62529, Saudi Arabia; amsfr@kku.edu.sa; 3 Department of Clinical Pharmacy, College of Pharmacy, King Khalid University, Abha 62529, Saudi Arabia; shahrani@kku.edu.sa; 4 Department of Pharmaceutics, Faculty of Pharmacy, King Abdulaziz University, Jeddah 21589, Saudi Arabia; elswaify2000@yahoo.com

**Keywords:** *Trichophyton rubrum*, eugenol, linalool, antifungal activity, emulgel

## Abstract

*Trichophyton rubrum* (*T. rubrum*) is the main cause of chronic dermatophytosis which is highly prevalent worldwide. This study was aimed to fabricate and characterize polymeric emulgel of eugenol and linalool for the treatment of *T. rubrum* infections. Using the slow emulsification method, the emulgel was prepared and characterized for thermodynamic stability, pH analysis, viscosity, spreadability, swelling behavior, %drug content, surface morphology, globules size, polydispersity index, surface charge (mV), thermal behavior, in vitro drug release and XRD studies. Biological activities of emulgel were conducted against *T. rubrum* in vitro and in vivo. Results indicated that emulgel formulations were thermodynamically stable. The pH of the formulations was within an acceptable range for skin. The viscosity and spreadability were optimum for the better patient compliance. The swelling behavior was 111.10 ± 1.25% after 90 min. The drug content was within the official pharmacopeia limit i.e., 100 ± 10%. The surface morphology revealed by scanning electron microscopy showed a spherical-shaped structure with characteristic larger cracks and wrinkles. The droplet size, PDI, and surface charge of the optimized emulgel were 888.45 ± 8.78 nm, 0.44 and −20.30 mV, respectively. The emulgel released 84.32% of eugenol and 76.93% of linalool after 12 h. There was complete disappearance of the diffraction peaks corresponding to the drugs after XRD analysis. In rabbits, the infection was safely and completely recovered after 12 days and the emulgel produced significant effects (*p* < 0.05) similar to the standard product Clotrim^®^. It is concluded that the eugenol–linalool emulgel best described all its physical properties and can be applied topically for the treatment of *T. rubrum* infections.

## 1. Introduction

Conventional semisolid dosage forms used for the topical application of drugs have several disadvantages. They may be sticky and have thick consistency. The patient feels difficulty in application of such kinds of ointments, lotions, and creams because sometimes they are applied on the skin by rubbing which produces severe discomfort. The stability of these preparations is another issue. To avoid these problems, polymer-based emulgels are now frequently used in the pharmaceutical and cosmetic industries [1].

Emulgel is a blend of polymer-based gel and emulsion which is beneficial in the sense that it is suitable for the delivery of hydrophobic and hydrophilic drugs across the skin into the blood stream, providing better release of the drugs [2]. Emulgels are preferred over other gels and simple emulsion for drug delivery because they are greaseless, thixotropic, emollient, easily applicable and removable, water soluble, non-staining, and have longer shelf life [3].

Eugenol is a slightly pale or colorless oily liquid which contains an aromatic ring and belongs to the allylbenzene class of the aromatic compounds [2,3]. It is naturally found in a variety of plants like *Cinnamomum tamala,* cinnamon, ginger, clove, dill, and star anise [4,5]. It is used as an antiseptic, anesthetic, antibacterial, and antifungal agent [6].

Linalool is a terpene alcohol naturally occurring in different plant flowers and spices. It has been estimated that more than 200 plant species are containing linalool. Lamiaceae, Rutaceae, and Lauraceae are the most famous plant families producing linalool in abundance [7]. Linalool has been obtained from the plant sources including *Artemesia vulgaris, Cananabis indica, Cannabis sativa, Cinnamomum tamala,* and *Humulus lupulus* [8]. Linalool, being an important ingredient of essential oil, is widely used for the treatment of joint pain. It has good analgesic activity, anxiolytic activity, anti-epileptic activity, and antifungal activity [9,10].

*T. rubrum* is a dermatophyte fungus belonging to the phylum Ascomycota and its favorite habitat is the outer-most dead layer of the skin, where it colonizes and causes severe infection. The surface of the colony while inside of the colony may appear yellow to brown and red in color. The growth of *T. rubrum* is quite slow, it appears as peg-shaped or tear drop-shaped, and its growth is inhibited by compounds containing nitrogen, sulfur, and phosphorus. The in vitro and in vivo studies have shown that its isolates have been reported to produce penicillin [9,10,11].

Infections caused by *T. rubrum* are usually transmitted by the use of contaminated garments, towels, and linens. *T. rubrum* attacks favorably on glabrous skin which is hair free [10]. Common infections like jock itch, athlete’s foot, ring worm, and fungal infection of nails and scalp are caused by *T. rubrum* [11]. *T. rubrum* infection is usually observed in the upper skin layer; however, deeper skin and muscles may also get infected by this fungus. It attacks the skin and may cause folliculitis, and in chronic cases it may cause the formation of granuloma of the skin especially in the patients of HIV [11].

Treatment of the fungal infection caused by *T. rubrum* totally depends upon the nature and severity of the infection. Miconazole, tolnaftate, clotrimazole and terbinafine are commonly available drugs for the treatment of *T. rubrum* infections. Topical preparations containing antifungal agents are usually used for the skin infections of *T. rubrum* however nails infections are not treated with the application of topical preparations due to the lack of absorption of drug from the surface of nail to nail plate [12].

The use of polymers in drug delivery systems or pharmaceutical dosage forms is considered safer particularly when they are intended for topical treatment of various microbial infections [13]. Carbomers are high-molecular-weight homo- and copolymers of acrylic acid cross-linked with a poly alkenyl polyether. They are used in pharmaceutical preparations as suspending agents, gel bases, emulsifiers, and binding agents. Carbomers, i.e., carbopols, are the most-used gelling agents in the manufacturing of creams, ointments, and gels meant for the topical application to the skin or mucous membranes [14]. They have long history of safe and effective use in semi-solid formulations.They has been demonstrated to have low irritancy and non-sensitizing properties with repeated usage [15].

Thus, this study was aimed to fabricate and characterize poly (acrylic acid)-based pharmaceutical emulgel co-loaded with eugenol–linalool for the treatment of *T. rubrum* infections.

## 2. Materials and Methods

### 2.1. Chemicals and Reagents

Carbopol 934 (Sigma Aldrich, Schnelldorf, Germany), Ethanol (Sigma Aldrich, Schnelldorf, Germany), Eugenol, Linalool (Sigma, CEDEX, St. Quentin Fallavier, France), PEG-400 (Sigma, Germany), Liquid paraffin (Biomed, India), Methanol (Bosch, Pakistan), Methyl parabene, Propyl paraben (Oxoid, UK), Span 20 (Himedia, India), Triethanolamine (Biomed, India) Tween 80 (Merck, Germany), Potato dextrose agar (PDA) (Oxoid, UK), Distilled water and Clotrim^®^ 1% cream (Clotrimazole) by Zafa Pharmaceutical Karachi Pakistan.

### 2.2. Experimental Animals

A local breed of male rabbits weighing of 1–2 kg were used in this study. The rabbits were purchased from the local market of Lahore and were put into steel cages. A bed of saw dust was applied uniformly in the cages. They were acclimatized for one week before the experiments and were provided free access to food and water ad libitum.

### 2.3. Preparation of Emulgel

The basic methodology for the formulation of an emulgel includes the preparation of an emulsion and then addition of a polymer as a gelling agent to form the emulgel. Emulgel was fabricated by the method reported by Burki et al. (2020), slightly modified [16]. The first step involved in the formulation of an emulgel is the preparation of an emulsion. For this purpose, the aqueous phase was prepared by making solution of tween 80 in the distilled water (Solution A). In the second step, methyl paraben and propyl paraben were dissolved in PEG-400 (Solution B). Both the solutions (A + B) were thoroughly mixed with continuous stirring. Oil phase was prepared by a mixing span of 20 in liquid paraffin (light). Eugenol and linalool in equal concentrations were also added to the oil phase. Both phases were separately heated at 70–75 °C and mixed using a mechanical stirrer (Daihan, Korea). The gel phase was formulated by using carbopol 934 which was soaked in distilled water overnight. It was then stirred continuously by using a mechanical stirrer and its pH was adjusted from 4.5 to 4.6 by using triethanolamine (TEA). When the dispersion of gelling agent was completed in the water and a well-dispersed mixture was formulated, it was ready for incorporation into the emulsion. Finally, the emulsion and gel were mixed in 1:1 ratio and constantly stirred until a uniform semi solid emulgel was obtained [2].

### 2.4. Evaluation and Characterizations of Emulgels

#### 2.4.1. Physical Examination and Stability

Emulgel formulations were physically examined for the evaluation of homogeneity, color, consistency, phase separations, and grittiness [16]. The formulations were subjected to heat and cool cycles, freeze–thaw cycles, and also kept at different temperatures as per ICH (International Council on Harmonization) guidelines for a time period of 28 days.

#### 2.4.2. Rheological Study of Emulgel

The viscosity of emulgel preparation was determined by using viscometer (NDJ, K8, Korea) at 25 °C. The readings were taken at specified time in triplicates and results were averaged.

#### 2.4.3. Spreading Coefficient

The spreading coefficient of the emulgel was determined using a specially modified apparatus in the laboratory by the drag and slip method. The apparatus was made of a wooden block with a mounted a pulley on its one edge, and a glass slide fitted on the wooden block. About 2 g of the emulgel was applied over the surface of glass slide mounted on the wooden block. Another glass slide with the same dimensions was taken and it was put over the fixed glass slide mounted on the wooded block in such a way that the emulgel was sandwiched between two glass slides. A 1 kg block was put on the glass the slide for 5 min to remove trapped air and to ensure uniform spreading of the emulgel. Then an 80 g weight was hung by attaching a thread to the top glass slide with the help of a hook. The thread was passed above the pulley and time was noted. The smaller the time taken by the slide to cover a distance of 7.5 cm on the fixed glass, the better the spreading coefficient of the preparation [16]. The following formula was used to determine the spreading coefficient.

Spreadability (S) = M × L ÷ T(1)

where,

S represents “Spreadability of formulation”.
M represents the “Weight tied to upper slide of the apparatus.
L represents the “Length of glass slides”.
and T represents the “Time taken to separate the slides from one another.

#### 2.4.4. Swelling Behavior

Swelling behavior of the emulgel was found by taking 1 g of the emulgel on aluminum foil (Porous). The foil containing emulgel was then put into a small beaker with 10 mL of 0.1 N NaOH solution. A small amount of sample from the aluminum foil was taken out from the beaker and dried at room temperature. It was then reweighed, and the swelling index was calculated by the following formula [17]:

(2) $$\mathrm{SI}\;=\;\frac{W2\;-\;W1\;}{W1}\;\times \;100$$

where,

SI indicates swelling index.
*W1* indicates original weight of emulgel.
*W2* indicates weight of swollen emulgel.

#### 2.4.5. Drug Content Determination

Drug content of the preparation was determined by using spectrophotometric method. Briefly, 1 g of the emulgel was taken in a test tube and mixed with 9 mL ethanol, centrifuged, and then filtered. The absorbance was recorded both for eugenol and linalool using UV-spectrophotometer (SHIMADZU, Kyoto, Japan). A standard solution was prepared in the same solvent and the absorbance was noted [16]. The drug contents were found using the following formula:

Drug Content = Concentration × Volume used × Dilution factor/Conversion factor(3)

#### 2.4.6. SEM Analysis

Analysis of surface morphology and apparent shape of emulgel was performed via scanning electron microscopy as reported in literature with slight modification [18]. The samples for SEM were prepared by attaching the emulgels on metal stubs with the help of double-sided adhesive tape and dried in a vacuum chamber. Then, a 10 nm thick gold coating was applied with a sputter-coater and observed under high resolution SEM.

#### 2.4.7. Globule Size, PDI, and Zeta Potential

The globule size (droplet size), PDI, and zeta potential of the prepared emulgel was tested by using Zetasizer (Malvern instrument, Great Malvern, UK) according to a study by Eid et al. (2014) slightly modified [19]. For this purpose, a small quantity of emulgel from each formulation was dispersed in distilled water and then a sample solution was injected into the Zetasizer to find the mean globule size of the emulgel.

#### 2.4.8. Thermal Behavior

Thermal behavior of the ingredients and emulgel was recorded by DSC and TGA. Under a nitrogen environment, a DSC of the ingredients and formulations was performed. Heat stress was gradually applied from ambient to 400 °C at a rate of 10 °C/min. TGA was conducted in a temperature range of 0–800 °C.

#### 2.4.9. In Vitro Drug Release

The in vitro release of eugenol and linalool from the emulgel was determined using Franz Diffusion Cell (IPS Technologies, New Delhi, India). This instrument consists of two compartments: a donor compartment and a receptor compartment. A skin or artificial membrane is usually fixed between these two compartments. The formulation is applied or loaded on the donor compartment and the receptor compartment is filled with buffer solution. The samples (1–3 mL aliquots) are withdrawn at specified period of time from the receptor compartment, and, at the same time fresh buffer is added to the receptor compartment to maintain the sink condition. The samples are then analyzed using UV visible spectrophotometer. In our study, 1 g of emulgel was applied on the donor compartment by fixing a cellulose membrane. The receptor compartment was filled with phosphate buffer solution pH 7.2. The samples were withdrawn at 0 h, 1 h, 2 h, 4 h, 8 h, and 12 h, and then analyzed by UV visible spectrophotometer (SHIMADZU, Japan).

#### 2.4.10. X-ray Diffraction Studies

The XRD studies of the ingredients and physical mixture, i.e., emulgel, were conducted by a Bruker AXS D-8 (Kista, Sweden) fitted with a CuK radiation detector. This was operated at a node voltage of 40 kV and the input current was 30 mA [20].

### 2.5. In Vitro Antifungal Activity

The optimized emulgel was then tested for its antifungal activity. The media used for the growth of fungi was potato dextrose agar (PDA) which was spread in a thin layer over the glass plate. One hundred microliters of fungal suspension was taken and poured on an agar plate and spread uniformly with the help of a sterile swab. A sterile filter paper disc of about 6 mm containing 50 μL of emulgel was placed on the agar plates. These plates were then placed in an incubator at 37 ± 2 °C. After 72 h, the zone of inhibition and clearance were calculated and recorded in mm. The blank formulation was used as a control in all the experiments. All the values were calculated in triplicates.

### 2.6. In Vivo Studies

#### 2.6.1. Ethics

The current study was approved by the institution ethical review board of Gomal University, D.I.Khan Pakistan under reference no. 506/QEC/GU.

#### 2.6.2. Experimental Design

The animals were divided into three groups containing five rabbits in each group (*n* = 5). Group-I was the control group which did not receive any treatment, Group-II was standard group treated with commercial product Clotrim^®^ (Zafa Pharmaceutical, Karachi Pakistan) containing clotrimazole (Standard group), andGroup-III was test/experimental group treated with prepared emulgel of eugenol and linalool (Experimental group).

#### 2.6.3. Fungal Strain

*T. rubrum* strain (ATCC) was provided by Microbiology Lab of University of Veterinary and Animal sciences Lahore Pakistan.

#### 2.6.4. Induction of Fungal Infection

Dermatophytosis was induced by the method of Ghannoum et al., (2016) with slight modification [21]. Briefly, rabbits were anaesthetized by intramuscular administration of an anesthetic combination of ketamine and xylazine (0.3 mL). An area of skin on the middle back of the rabbits was clipped and shaved and a 6.25 cm^2^ area was abraded with sandpaper. About 0.1 mL suspension containing 1 × 10^7^ cells of *T. rubrum* was applied to the marked area using a sterile pipette-tip and rubbed thoroughly.

#### 2.6.5. Clinical Evaluation and Scoring of Infection

The treatments were repeated each day and continued for 12 days. The lesions were examined continuously from 1 day post-infection to 12 days post-infection [22]. Clinical evaluations were done according to the severity of induced infection and each area was scored from 0 to 5.

### 2.7. Statistical Analysis

The SPSS (IBM, Version 20) and EXCEL were used as statistical tool. Data were expressed as mean ± SEM. Student t test and one way ANOVA were applied to data set.

## 3. Results and Discussion

### 3.1. Physical Properties of Eugenol–Linalool Emulgel

In preparation of any emulgel, the physical properties of emulsion are mostly considered and the preparation which has high consistency, elegance, and stability is preferred [22]. The results of organoleptic studies indicated that the preparation best described its physical characteristics. Physical properties of the formulation were observed for 28 days (A = at 8 °C, B = at 25 °C, C = at 40 °C, D = at 40 °C ± 75% RH) and recorded. The color of the formulation remained white throughout the time span of evaluation. Similarly, no smell was observed from the formulation at any temperature. The excellent homogeneity was observed for all the formulations. No phase separation was observed at all in the formulation. Similarly, no grittiness was observed at all in the formulations at any temperature at any time interval. The details are shown in the Table 1.

### 3.2. Viscosity of Prepared Emulgels

Viscosity plays an important role in the delivery of drugs used via topical or transdermal application. Several characteristics of formulations, including the stability, spreadability, drug release, and ease of application, are dependent on the viscosity [18]. The viscosity of the formulations may also be influenced by various polymers used as gelling agents (e.g., carbopol), surfactants and co-surfactants, different oils, and co-solvents used in the formulations [16]. The viscosities of the ELE2 formulation determined at different times and temperatures are given in Table 2. There was some variation in the viscosities of ELE2 formulation kept at different temperatures; however, it was insignificant, i.e., *p* > 0.05.

### 3.3. Evaluation of Spreadability

The extent to which a topically applied pharmaceutical formulation is spread on skin is termed as its spreadability [16]. It is considered the fundamental characteristic of topical formulations upon which the therapeutic efficacy depends [23]. Formulation having optimum spreadability requires a small shear to come out of a container [17]. Various factors including concentrations of polymers and low and elevated temperatures affect the spreadability coefficient of topical formulation. Indeed, at low temperatures, the viscosity of the formulation increases which results in low spreadability. Similarly, at elevated temperatures, the viscosity of the topical formulations decreased, resulting in high spreadability. Average spreadability values for blank formulation (ELE1), and drug-loaded formulation (ELE2) have been presented in Table 3.

### 3.4. Swelling Index of Emulgels

The action of the polymer in pharmaceutical preparation is enhanced when the macromolecule is fully swollen. The swelling provides rheology modification, suspending properties, and emulsification to the topical formulations [16]. Polymer swelling can be accomplished in several ways, including neutralization or hydrogen bonding [15,16]. The swelling index of a blank formulation (ELE1) and a drug-loaded emulgel (ELE2) formulations were tested. It was observed that ELE1 showed swelling indexes of 113.33 ± 1.14, 119.33 ± 1.17%, and 121.56 ± 1.20%, at time intervals of 30, 60, and 90 min, respectively. ELE2 showed swelling indexes of 88.880 ± 1.18%, 100.50 ± 1.21, and 111.10 ± 1.25 at time intervals of 30, 60, and 90 min, respectively. Results are shown in Table 4.

### 3.5. Drug Content Analysis

The uniform distribution of drugs in any pharmaceutical preparation is important for its therapeutic effect and can be confirmed by percent of drug content [19]. The results indicated that 91.34 ± 1.87% of eugenol and 92.29 ± 1.73% of linalool were obtained after analysis. There was an insignificant difference (*p* > 0.05) between the percent drug content for both drugs. The results of the drug content revealed that %drug content was within the official limit (i.e., 100 ± 10%) allowed by the United States Pharmacopeia (USP) [16].

### 3.6. SEM Analysis

**Figure 1:**
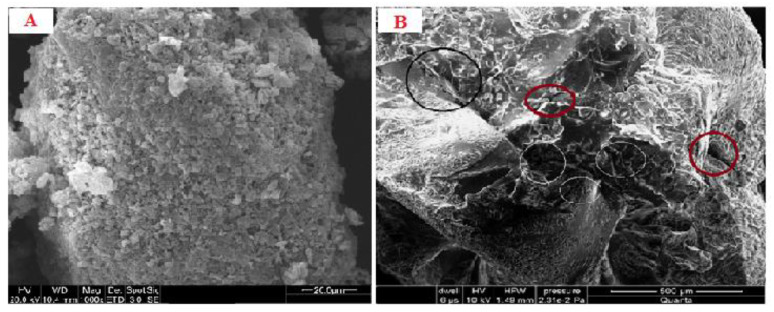
(**A**) SEM micrograph of blank emulgel (**B**) SEM micrograph of drug-loaded emulgel.

The SEM analysis of the emulgel revealed an almost spherical-shaped structure with characteristic large wrinkles and cracks distributed throughout the surface (Figure 1A,B). The drug-loaded formulation displayed a tight outer surface that possessed small pores or channels, which were possibly created as a result of the migration of water molecules during the drying process and consequent contraction of the polymer networks. The slight shape distortion was perhaps due to larger differential shrinkage as water was evaporated. The surface displayed numerous oil pockets of uneven size, which could be due to the coalescence of oil droplets during external cross-linking process [20].

### 3.7. Droplet Size, PDI, and Zeta Potential

**Figure 2:**
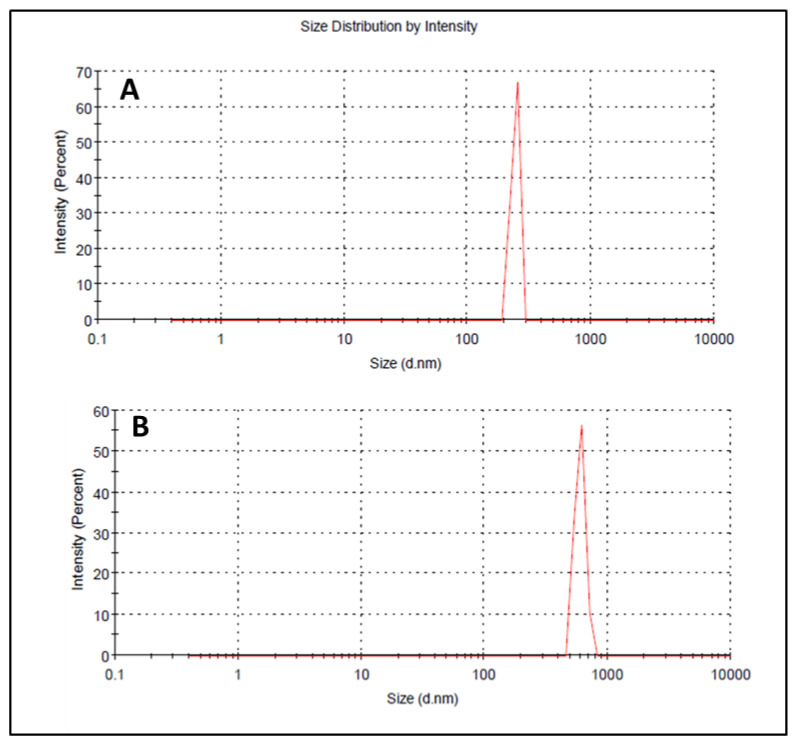
(**A**) Size distribution and PDI of ELE 1 and (**B**) ELE 2 formulations.

**Figure 3:**
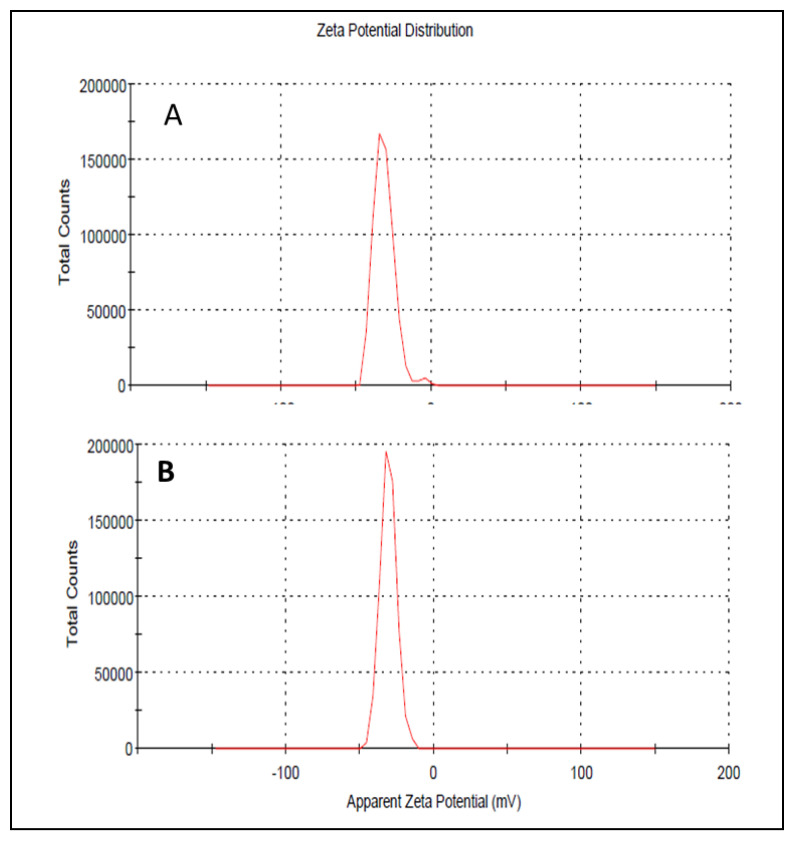
(**A**) Zeta potential distribution of ELE 1 and (**B**) ELE 2 formulations.

Droplet size has an important role in the formulations used for topical or transdermal delivery of drugs [20]. Several promising parameters like drug release, drug permeation, and bio-distribution are affected by the droplet size and the particle size distribution (PSD) [21]. ELE1 has droplet size of 470.84 ± 7.30 nm (Table 5 and Figure 2A) which was significantly lower as compared to that of ELE2. The droplet size of the ELE2 has increased after loading of drugs (888.45 ± 8.78) as shown in Figure 2B. Our data agreed with Sarin et al., (2018) who reported that addition of curcumin to a blank formulation increased the droplet size from 178.7 ± 1.4 nm to 183.6 ± 1.7 nm [22]. PDI indicates the uniformity and homogeneity of droplet size or size distribution in the formulations. The higher the PDI, the lower the uniformity of droplet size/size distribution in the formulation will be, and vice versa [20]. The formulation having PDI less than 0.45 is considered a homogeneous dispersion. The PDI values of ELE1 and ELE2, were 0.135 and 0.44, respectively, indicating the uniformity of droplet/globule size within each formulation. The surface charges (mV) of ELE1 and ELE2 were negative (i.e., −23.30 mV and −20.30 mV, respectively) on their surfaces due to the addition of carbopol to the formulations, because carbopol is a highly negatively-charged polymer. The zeta potential distribution of both formulations is presented in Figure 3. The negative zeta potential is beneficial for enhanced stability of the formulations [18]. According to studies conducted by Eid et al. [19] and Kumar et al. [20], formulations with negative or positive zeta potential, i.e., surface charges, have enhanced stability. This is because of the increased electrostatic repulsion between the droplets or globules of the emulsion system and thus it will prevent droplet coalescence [19,20].

### 3.8. Thermal Analysis

**Figure 4:**
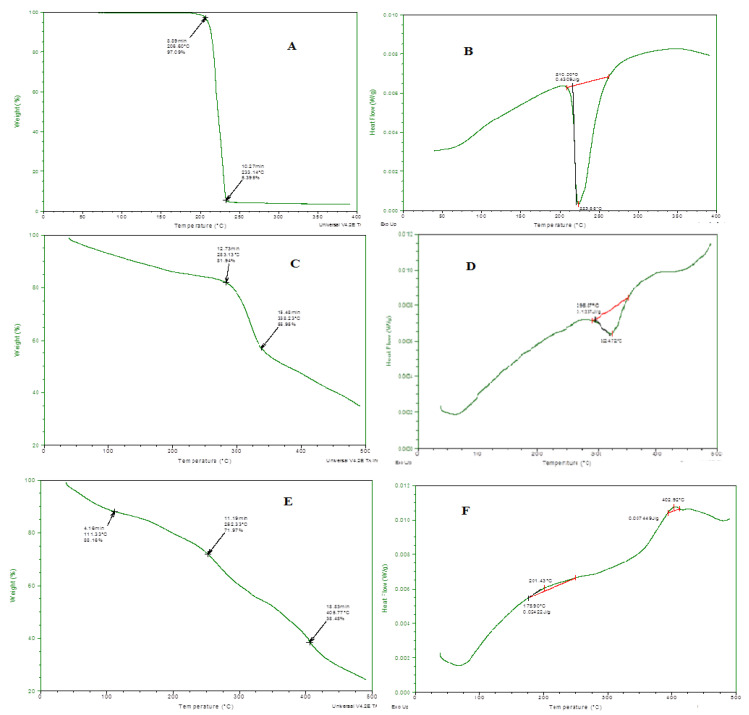
(**A**) TGA spectra of linalool, (**B**) DSC spectra of linalool, (**C**) TGA Spectra of eugenol, (**D**) DSC spectra of eugenol, (**E**) TGA spectra of ELE2, (**F**) DSC of ELE2.

Thermal stability of all the individual ingredients and prepared emulgel was analyzed through thermo-gravimetric and DSC measurements, and results are presented in Figure 4. In the TGA curve, initial weight loss indicates the loss of moisture content at various temperatures. When the degradation pathway of emulgel was studied, it showed more thermal stability even at higher temperatures, i.e., 400 °C, 65% loss of mass occurred. Through DSC, glass transition temperature of the prepared formulation was also measured. A clear difference was observed in a DSC thermogram of individual ingredients and drug-loaded formulations. This shifting of peaks toward a higher temperature also confirmed the grafting and development of rigid polymeric network systems [18].

### 3.9. In Vitro Drug Release

**Figure 5:**
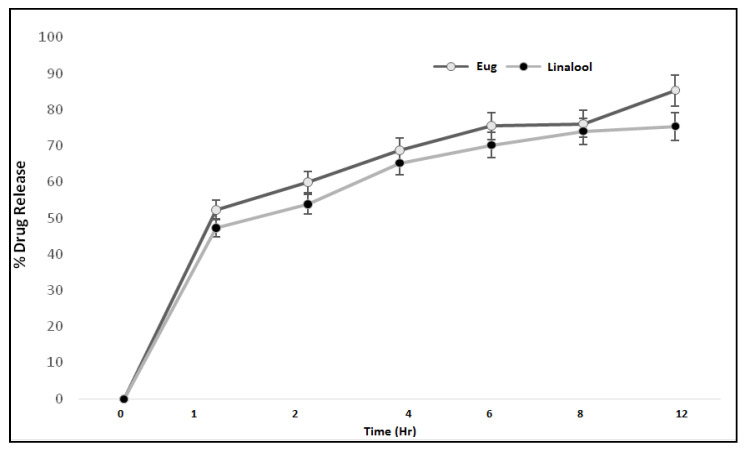
Percent drug release of eugenol and linalool from ELE2 at different time intervals.

The therapeutic efficacy of any drug depends upon the release of drug from pharmaceutical dosage forms [21]. The release of drug from a topical formulation is dependent upon several factors including gelling agents (polymers), emulsifying agents, spreadability, and viscosity [22]. The release of eugenol and linalool from an emulgel formulation (ELE2) is presented in Figure 5. The percent drug release of eugenol was observed as 0%, 51.58%, 58.9%, 69.80%, 76.5%, 77.11%, and 84.32% at different time intervals. While the release of linalool from the ELE2 formulation was noted as 0%, 46.69%, 54.8%, 66.21%, 71.20%, 75.30%, and 76.93%. There is an insignificant difference (*p* > 0.05) between % release of drugs, i.e., eugenol and linalool from ELE2 formulation. The lower drug release from the formulation coukd be correlated to the presence of a gelling agent which makes a complex gel network and thus caused longer diffusion pathways of drug permeated through the membrane [23]. The same results were reported by Yen et al. (2015) who stated that drug release is inversely proportional to the amount of gelling agent added to the formulation [23].

### 3.10. XRD Studies

**Figure 6:**
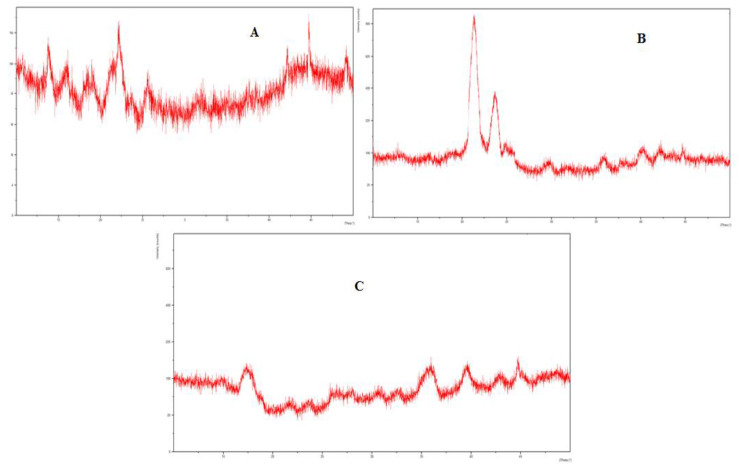
(**A**) XRD spectra of linalool, (**B**) XRD spectra of eugenol, (**C**) XRD spectra of emulgel (Physical mixture).

The XRD analysis of eugenol, linalool, and the physical mixture, i.e., emulgel was carried out to evaluate the solid-state transformation. The PXRD chart of eugenol and linalool displayed characteristics peaks at 11.7°, 15.7°, 17.2°, 21.4°, 22.5°, 23.6°, 30.2°, and 43.6°. These sharp and intense peaks demonstrate the crystalline nature of formulation. The distinctive peaks of the eugenol and linalool were not detected in the emulgel formulation. There was a complete disappearance of diffraction peaks corresponding to the eugenol and linalool portrayed by the PXRD patterns of drug-loaded emulgel. The results of the XRD analysis are presented in Figure 6.

### 3.11. Biological Evaluation of the Prepared Emulgel

#### 3.11.1. In Vitro Anti-Fungal Activity

**Figure 7:**
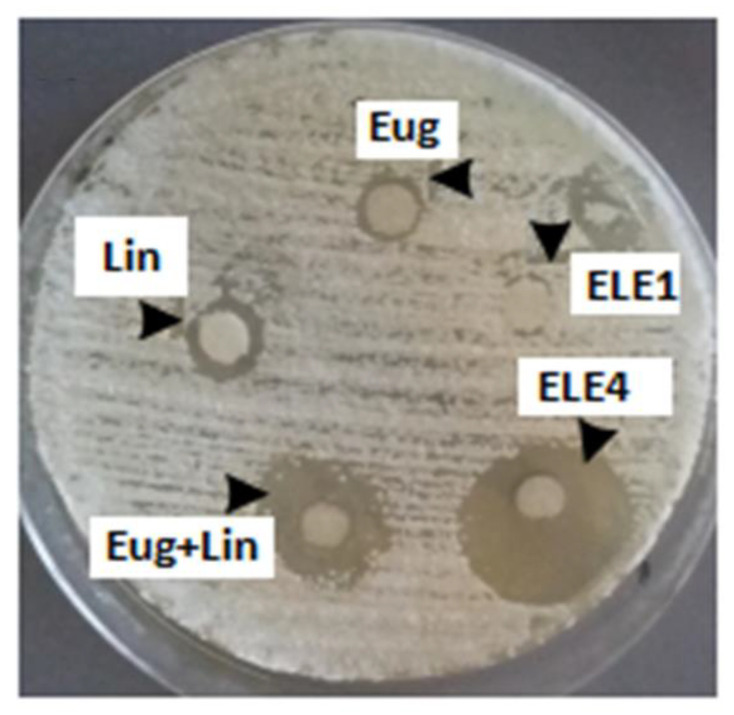
Zone of inhibitions of eugenol, linalool, eugenol + linalool, ELE1 and ELE2.

The results of in vitro antifungal activity indicated that when eugenol and linalool were applied in the quantity of 50 μL, the growth of fungus was inhibited by 2.5 mm and 3.5 mm, respectively. Similarly, eugenol and linalool were used in combination against the *T. rubrum* and the zone of inhibition was 5.5 mm. The blank formulation did not show any antifungal activity while eugenol–linalool-loaded emulgel (ELE2) formulation showed significant (*p <* 0.05) antifungal activity by inhibiting the zone (10.5 mm) of *T. rubrum* as compared to the pure eugenol and linalool. The detailed results are shown in Table 6 and Figure 7. The results showed that the growth of *T. rubrum* was inhibited by eugenol and linalool. Linalool was more effective in growth inhibition of *T. rubrum* as compared to eugenol. It was also observed that co-loaded emulgel of eugenol and linalool significantly (*p <* 0.05) inhibited the growth of *T. rubrum.*

Thus, it can be concluded that the emulgel prepared by using a combination of linalool and eugenol is more effective in inhibition of *T. rubrum* growth.

#### 3.11.2. Induction of Infection

In vivo studies were performed on rabbits and they were divided into three groups. First, infection was induced on the back of animals and the animals were observed for 72 h for the assessment of induction of infection. It was observed that maximum infection was induced in 72 h in all the groups treated with fungal strain. Details are shown in Table 7.

#### 3.11.3. Assessment of In Vivo Antifungal Activity

**Figure 8:**
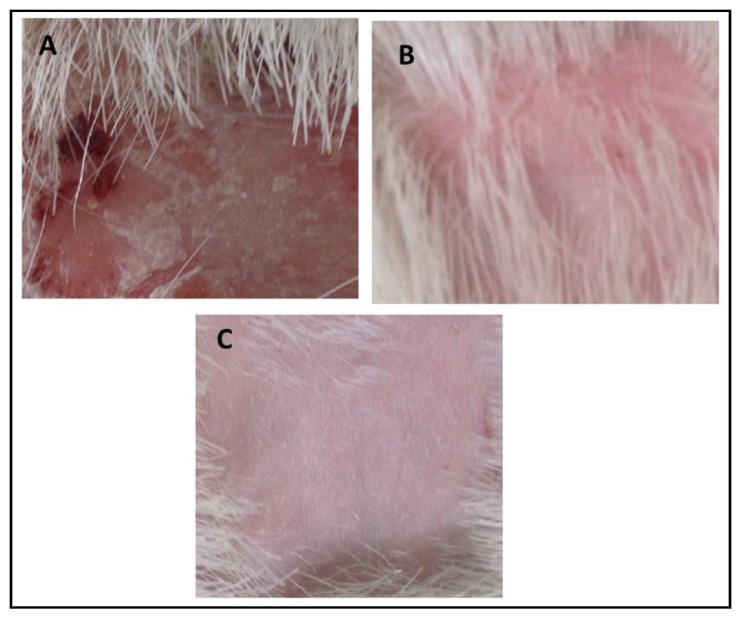
(**A**) Control group, (**B**) recovery from infection in the standard group, (**C**) recovery from infection in the experimental group.

In vivo antifungal activity was performed using rabbits as experimental animals after induction and confirmation of fungal infections. The results indicated that no recovery from infection was observed in Group-1 animals which were kept as a control group, and were given only a normal routine diet. The relative score of 5 was observed in Group-II (Standard group) animals which indicated that extensive damage to the skin and complete hair loss were observed in the rabbits. In the first three days, partial damage to the skin and hair loss was observed while in the next three days large areas of redness, scaling, exposed patches, and ulceration were observed, while in the next four days redness, swelling, bald patches, and scaly areas were seen. After treatment with marketed cream Clotrim^®^ for 12 days, the infection was safely and completely recovered, and the relative score reached 0. It cured the infection in 10–12 days. Group-3 was treated with eugenol–linalool emulgel which cured the infection in 12 days. Thus, the emulgel produced results quite similar to those of the standard group. The fungal infections were recovered only if a large amount of active ingredients was released by the pharmaceutical dosage form that penetrates skin layers and was retained for a prolonged duration at the site of infection [24]. According to the Zhou et al. (2016), the killing or eradication of fungus and fungal infections may be attributed to the smaller droplet size of the pharmaceutical preparations that allow high permeability of active moiety, and subsequently form a drug depot beneath the stratum corneum of the skin [25]. The details of the in vivo antifungal activity are shown in Table 8 and Figure 8.

## 4. Conclusions

This study concludes that the poly (acrylic acid)-based pharmaceutical emulgel co-loaded with eugenol and linalool for the treatment of *T. rubrum* infections is highly stable and the physical properties of the emulgel remained feasible over an extended period of time. It is also concluded that the linalool and eugenol emulgel best described all its physicochemical features and can be safely applied topically to treat infections caused by *T. rubrum.*

## Figures and Tables

**Table 1:** Physical properties of eugenol–linalool emulgel noted.

		Freshly Prepared	After 12 h	After 24 h	After 7 Days	After 14 Days	After 28 Days
Color	A	WC	WC	WC	WC	WC	WC
	B	WC	WC	WC	WC	WC	WC
	C	WC	WC	WC	WC	WC	WC
	D	WC	WC	WC	WC	WC	WC
Smell	A	-ve	-ve	-ve	-ve	-ve	-ve
	B	-ve	-ve	-ve	-ve	-ve	-ve
	C	-ve	-ve	-ve	-ve	-ve	-ve
	D	-ve	-ve	-ve	-ve	-ve	-ve
Homogeneity	A	***	***	***	***	***	***
	B	***	***	***	**	***	**
	C	***	***	***	**	***	**
	D	***	***	***	***	***	***
Phase separation	A	-ve	-ve	-ve	-ve	-ve	-ve
	B	-ve	-ve	-ve	-ve	-ve	-ve
	C	-ve	-ve	-ve	-ve	-ve	-ve
	D	-ve	-ve	-ve	-ve	-ve	-ve
Grittiness	A	-ve	-ve	-ve	-ve	-ve	-ve
	B	-ve	-ve	-ve	-ve	-ve	-ve
	C	-ve	-ve	-ve	-ve	-ve	-ve
	D	-ve	-ve	-ve	-ve	-ve	-ve

WC = white color, -ve = No phase separation, no smell and grittiness found, *** = Excellent rating for homogeneity, ** = Good rating for homogeneity, A = at 8 °C, B = at 25 °C, C = at 40 °C, D = at 40 °C ± 75% RH.

**Table 2:** Viscosities (Centipoise) of ELE2 at the indicated temperature and time.

Time	Viscosities at 8 °C	Viscosities at 25 °C	Viscosities at 40 °C
Day 0	14,390	14,390	14,390
Day 1	14,380 ± 13.7	14,310 ± 13.1	13,101 ± 10.2
Day 2	14,380 ± 12.5	14,200 ± 13.3	12,990 ± 11.3
Day 7	14,350 ± 13.2	14,120 ± 13.7	12,000 ± 11.3
Day 14	14,342 ± 13.9	14,100 ± 12.2	11,970 ± 11.5
Day 28	14,337 ± 12.4	13,980 ± 13.2	11,950 ± 13.2

All the values are calculated as Mean ± SEM; ELE2: Drug-loaded emulgel.

**Table 3:** Average spreadability values of ELE 1 and ELE 2 kept at the indicated temperature.

Formulations	Spreadability at 8 °C	Spreadability at 25 °C	Spreadability at 40 °C
ELE 1	16.90 ± 1.11	20.72 ± 1.35	26.43 ± 1.98
ELE 2	16.89 ± 1.17	20.77 ± 1.10	26.00 ± 11.2

All the values are calculated as Mean ± SD.

**Table 4:** Swelling index of blank (ELE1) and drug-loaded (ELE2) formulations.

Formulation Code	Time (Min)	Swelling Index ± SD (%)
ELE1	30 60 90	113.33 ± 1.14% 119.33 ± 1.17% 121.56 ± 1.20%
ELE2	30 60 90	88.880 ± 1.18% 100.50 ± 1.21% 111.10 ± 1.25%

**Table 5:** Droplet size, zeta potential, and PDI of ELE1 and ELE2 formulations.

Formulations	Droplet Size ± SD (nm)	Zeta Potential ± SD (mV)	PDI Ratio
ELE1 *	470.84 ± 7.30	−23.30	0.135
ELE2	888.45 ± 8.78	−20.30	0.44

* indicates: Blank formulation.

**Table 6:** Effect of linalool, eugenol and linalool-eugenol emulgel on growth of *T. ruburum*.

S.No	Treatment	Zone of Inhibitions
1	Eugenol	2.5 ± 0.12 mm
2	Linalool	3.5 ± 0.14 mm
3	Eugenol + Linalool	5.5 ± 0.19 mm
4	Blank formulation (ELE1)	0 mm
5	Drug-loaded formulation (ELE4)	10.5 ± 0.8 mm

**Table 7:** Induction of infection in different groups of rabbits.

S.No	Groups	Induction of Infection			
		0 h	24 h	48 h	72 h
1	Control group	0	0	2	4
2	Standard group	0	0	2	4
3	Experimental group	0	0	2	4

0 indicates no infection; 1 is slightly erythematous patches. 2 shows redness, bald patches, swelling with bristling hairs and scaly areas. 3 shows large areas of marked redness and ulceration. 4 indicates partial damage to the skin and hair loss. 5 indicates extensive damage to the skin and complete hair loss.

**Table 8:** Relative scoring after treatment of rabbits for 12 days.

Days	Group-1	Group-11	Group-III
	Control Group	Standard Group	Experimental Group
Day1	4	4	4
Day2	5	4	2
Day3	5	4	3
Day4	5	3	3
Day5	5	3	3
Day6	5	3	2
Day7	5	2	1
Day8	5	1	1
Day9	5	1	1
Day10	5	0	1
Day11	5	0	0
Day12	5	0	0

## Data Availability

It can be obtained from the corresponding author on request.
